# Environmental change, shifting distributions, and habitat conservation plans: A case study of the California gnatcatcher

**DOI:** 10.1002/ece3.3482

**Published:** 2017-10-28

**Authors:** Heather L. Hulton VanTassel, Michael D. Bell, John Rotenberry, Robert Johnson, Michael F. Allen

**Affiliations:** ^1^ University of California Riverside CA USA; ^2^ Department of Biology University of California Riverside CA USA; ^3^ Center for Conservation Biology University of California Riverside CA USA; ^4^ U.S. Department of Interior Air Resources Division National Park Service Lakewood CO USA; ^5^ Present address: Department of Ecology, Evolution, and Behavior University of Minnesota Saint Paul MN USA; ^6^ Present address: The Nature Conservancy 1417 Engals Blvd., Suite #100 Mt. Pleasant SC 29464

**Keywords:** California gnatcatcher (*Polioptila californica*), climate change, conservation lands, ecological niche models, habitat conservation plans, land‐use change

## Abstract

Many species have already experienced distributional shifts due to changing environmental conditions, and analyzing past shifts can help us to understand the influence of environmental stressors on a species as well as to analyze the effectiveness of conservation strategies. We aimed to (1) quantify regional habitat associations of the California gnatcatcher (*Polioptila californica*); (2) describe changes in environmental variables and gnatcatcher distributions through time; (3) identify environmental drivers associated with habitat suitability changes; and (4) relate habitat suitability changes through time to habitat conservation plans. Southern California's Western Riverside County (WRC), an approximately 4,675 km^2^ conservation planning area. We assessed environmental correlates of distributional shifts of the federally threatened California gnatcatcher (hereafter, gnatcatcher) using partitioned Mahalanobis *D* ^2^ niche modeling for three time periods: 1980–1997, 1998–2003, and 2004–2012, corresponding to distinct periods in habitat conservation planning. Highly suitable gnatcatcher habitat was consistently warmer and drier and occurred at a lower elevation than less suitable habitat and consistently had more CSS, less agriculture, and less chaparral. However, its relationship to development changed among periods, mainly due to the rapid change in this variable. Likewise, other aspects of highly suitable habitat changed among time periods, which became cooler and higher in elevation. The gnatcatcher lost 11.7% and 40.6% of highly suitable habitat within WRC between 1980–1997 to 1998–2003, and 1998–2003 to 2004–2012, respectively. Unprotected landscapes lost relatively more suitable habitat (−64.3%) than protected landscapes (30.5%). Over the past four decades, suitable habitat loss within WRC, especially between the second and third time periods, was associated with temperature‐related factors coupled with landscape development across coastal sage scrub habitat; however, development appears to be driving change more rapidly than climate change. Our study demonstrates the importance of providing protected lands for potential suitable habitat in future scenarios.

## 1 INTRODUCTION

Anthropogenic climate change is known to significantly impact biodiversity (Dukes & Mooney, 1999; Sala et al., 2000; Vitousek, DAntonio, Loope, Rejmanek, & Westbrooks, 1997) by fragmenting, shifting, increasing, and/or decreasing geographic distributions of many species (Pearson & Dawson, 2003; Peterson, Schreiner, & Buckingham, 1997). Together, these changes in geographic distributions can lead to the local or regional extinction of species (Thomas et al., 2004) or the generation of novel communities (Hobbs et al., 2006; Ohlemüller, Walker, & Wilson, 2006; Williams & Jackson, 2007). Furthermore, there are multiple environmental factors that can influence a species’ distribution, and recent research has shown that a species’ ability to respond to climate change may be affected by multiple natural and anthropogenic factors (e.g., Brook, Sodhi, & Bradshaw, 2008; Preston, Redak, Allen, & Rotenberry, 2012; Swab, Regan, Keith, Regan, & Ooi, 2012).

Ecological niche modeling can provide insights into consequences of environmental change on biodiversity (Barrows & Murphy‐Mariscal, 2012; Preston et al., 2012; Thomas et al., 2004), and it is increasingly used to evaluate the responses of biodiversity to changes in climate and other environmental attributes (e.g., Dawson, Jackson, House, Prentice, & Mace, 2011; Pereira et al., 2010; Preston et al., 2012; Thomas et al., 2004). Results from niche models can be used to plan for priority area selection for conservation purposes, and many habitat conservation plans created to mitigate climate and environmental change are based on future geographic ranges (summarized in Peterson, 2006). However, there can be substantial uncertainty when predicting future species’ distributions due to the uncertainty of future climatic conditions, potential patterns of landscape development, changing species interactions, and even species’ adaptations to new conditions. Nevertheless, many species have already undergone shifts in distribution due to habitat and climatic changes, and we can use past shifts to better understand the influence of environmental factors on a species’ range and to evaluate, create, and/or adapt habitat conservation plans, to the extent that past geographic shifts show us the trajectory of change. Furthermore, analyzing past shifts may provide us with the ability to evaluate the effectiveness of existing conservation protections.

**Figure 1:**
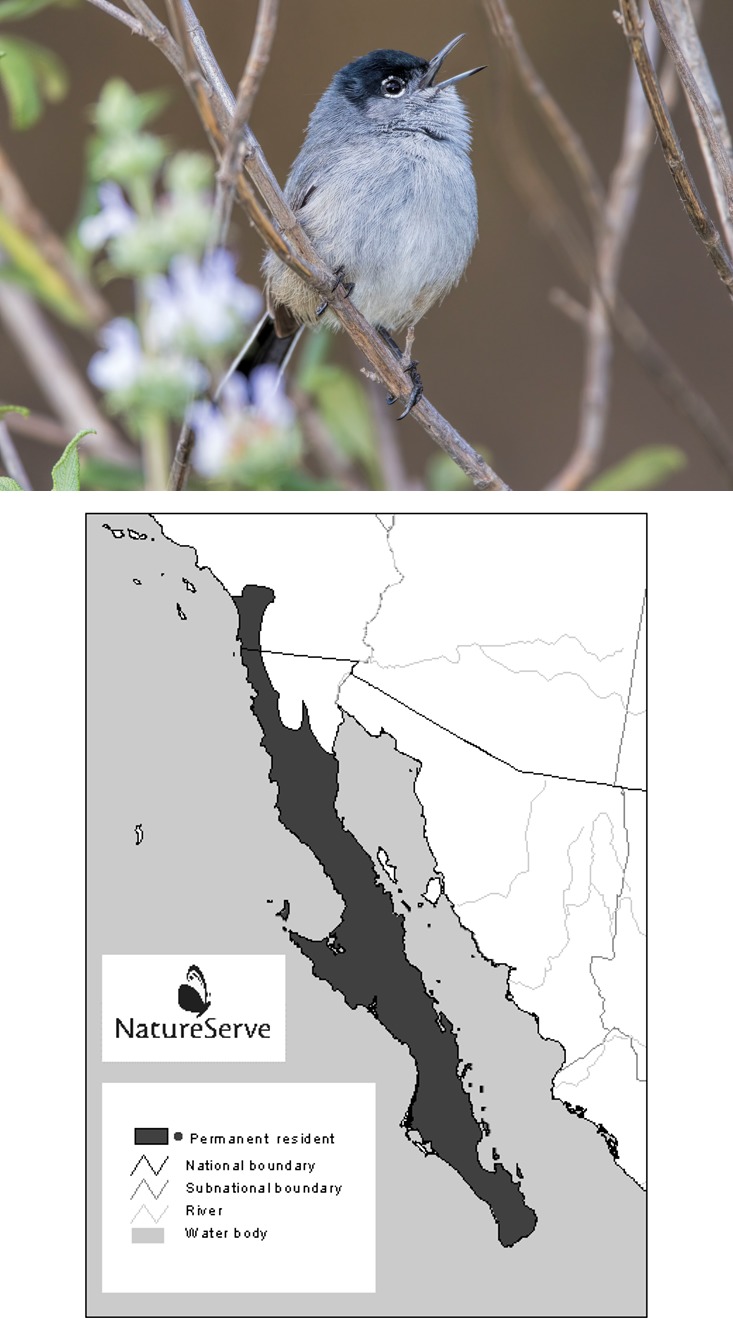
(Upper) California gnatcatcher (*Polioptila californica*) and (lower) its geographic range. Map adapted from birdphotos.com with original data acquired from Ridgely et al. (2005). Photo courtesy of Mark A. Chappell

Southern California is one of the world's biodiversity hotspots (Myers, Mittermeier, Mittermeier, da Fonseca, & Kent, 2000). However, this region continues to experience a multitude of anthropogenic disturbances, including but not limited to urbanization, air pollution, the introduction of non‐native species (summarized in Lovich & Bainbridge, 1999), and climate change. We analyzed past habitat associations and distributional shifts of the federally threatened California gnatcatcher (*Polioptila californica;* hereafter gnatcatcher; see Figure 1 for gnatcatcher photo) during different time periods within southern California's Western Riverside County (WRC) planning area using ecological niche modeling. We chose the gnatcatcher as a case study due to an extensive observation and survey database and because it is embedded in a regional conservation plan for WRC. The gnatcatcher‐preferred vegetation type is coastal sage scrub (CSS; Atwood, 1993). CSS communities are among the most endangered habitats in the United States with estimated losses of 60%–90% since the start of the 20th century (O'Leary, 1995). Within the remaining intact CSS, many areas have been invaded by exotic grasses (Minnich & Dezzani, 1998) through the combination of nitrogen deposition from urban southern California (Allen, Padgett, Bytnerowicz, & Minnich, 1998; Padgett & Allen, 1999) and fire (Cox, Preston, Johnson, Minnich, & Allen, 2014). As shrub cover and shrub species diversity decline, critical CSS habitat for more than 200 plant and animal species that are currently endangered, threatened, or of “special concern” is compromised (Bowler, 2000).

To better understand the influence of past environmental changes and the importance of conservation lands in fulfilling a mandate to protect this species, we had four aims: (1) to quantify regional habitat associations of the gnatcatcher; (2) to describe changes in environmental variables and gnatcatcher distributions through time; (3) to identify environmental drivers that are associated with habitat suitability changes; and (4) to relate habitat suitability gains and losses to habitat conservation plans. Because our analyses covered pre‐ and postconservation planning periods, we expected to see shifts in the distribution of gnatcatchers associated with overall deteriorating environmental conditions that helped trigger conservation action in the first place. As there are many known environmental stressors within WRC, we expected to see a suite of environmental variables as drivers for shifting distributions. Nevertheless, we anticipated that urbanization would be a stronger driver than climatic variables due to greater pace of change of the former compared to the latter. We also expected that acquired conservation lands would help mitigate the negative effects of environmental change.

## 2 METHODS

**Figure 2:**
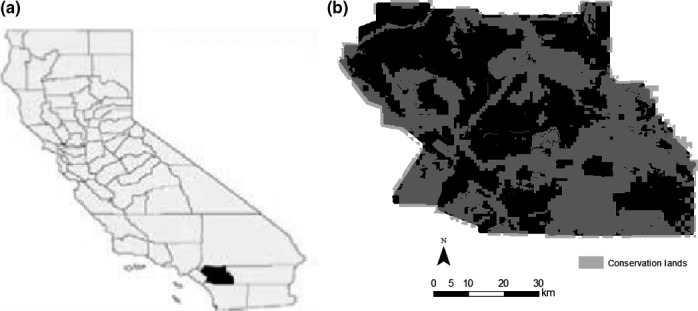
(a) Map of California's counties. Our study site, Western Riverside County, is highlighted in black. (b) Map of conservation lands as of 2012 within WRC highlighted in gray

Our study location was southern California's WRC (Figure 2), at the northern extent of the California gnatcatcher's geographic range (Figure 1). Rapid urban development beginning in the 1970s, overlaid on extensive agricultural development that started in the late 1800s, led WRC to develop one of the first multiple species habitat conservation plans in the state in 2004 (planning initiated in 1997, plans established by 2004) to protect a growing number of species, including the gnatcatcher, listed as rare, threatened, endangered, or sensitive by both the state and federal governments (Preston & Rotenberry, 2007; Preston, Rotenberry, Redak, & Allen, 2008; Western Riverside County 1997). We divided the ~4,675‐km^2^ study area into a grid of 74,832 250 m × 250 m cells. Each cell contained values of environmental attributes described in the following sections and in Table 1. We compiled presence data for the gnatcatcher from multiple sources, including online databases, government databases, museum records, published and unpublished accounts, environmental impact reports, and field notes of local naturalists. The largest sources of records are surveys conducted by Center for Conservation Biology (CCB) and the University of California, Riverside (UCR) biologists over the last decade within WRC. Not all location data are freely available due to US Fish and Wildlife Service restrictions; data that are available may be found in the California Natural Diversity Database (https://www.wildlife.ca.gov/Data/CNDDB/Maps-and-Data). We only used records with spatial precision of <125 m. We deleted all spatially redundant records (observations within the same grid cell) for each time period independently.

**Table 1:** Environmental variables included in the niche models and subsequent analyses. The first 12 environmental variables were used in the ecological niche modeling. All environmental variables were included in additional analyses

Variable	Variable type	Description
*Minimum Temperature*	Climatic	Average minimum temperature for month of January. Values stored in raster with 1‐km resolution (°C)
*Maximum Temperature*	Climatic	Average maximum temperature for month of July. Values stored in raster with 1‐km resolution (°C)
*Precipitation*	Climatic	Average annual precipitation (mm)
*Elevation*	Topographic	Landscape‐scale representation of elevation above mean sea level for an 8 × 8 neighborhood at 30‐m resolution (m)^a^
*Aspect (North Facing)*	Topographic	Local‐scale representation of northern aspect for an 8 × 8 neighborhood at 30‐m resolution (Domain −0.999 to 0.998)^a^
*Aspect (East Facing)*	Topographic	Local‐scale representation of eastern aspect for an 8 × 8 neighborhood at 30‐m resolution (Domain −0.999 to 0.998)^a^
*Slope*	Topographic	Local‐scale representation of slope for an 8 × 8 neighborhood at 30‐m resolution (° above horizontal)^a^
*Coastal Sage Scrub*	Vegetation	Local‐scale representation of percent coastal sage scrub land cover
*Chaparral*	Vegetation	Local‐scale representation of percent chaparral land cover
*Grassland*	Vegetation	Local‐scale representation of percent grassland cover
*Agricultural Land*	Other Stressor	Local‐scale representation of percent agriculture land use
*Developed Land*	Other Stressor	Percent of urban development in the landscape
*High Exotic Cover*	Other Stressor	Percent of the CSS with >25% Exotic Cover
*Fire History*	Other Stressor	Number of fires that occurred over a 60 year time period (1943–2003)
*Total Nitrogen Deposition*	Other Stressor	Total amount of nitrogen in soil (ppm)

a 8 × 8 neighborhood at 30‐m resolution is approximately equivalent to a 250 m × 250 m grid cell.

We split the observational data into three time periods: (1) 1980–1997, (2) 1998–2003, and (3) 2004–2012. Dates were chosen to represent both land management and ecological changes in WRC. The first time period was essentially preconservation planning. Native habitat, especially CSS, began rapidly disappearing due to conversion to urban and suburban development and expanding agricultural development beginning in the mid‐1980s (O'Leary, 1995). Concern for these habitat losses coupled with the federal listing of the gnatcatcher as threatened in 1993 triggered the second phase where planning for the creation of the WRC Multiple Species Habitat Conservation Plan (WRCMSHCP) began in 1998 (Western Riverside County 1997). This led to extensive new surveys of a wide range of species throughout the region, including by the CCB from 1998 to 2004. The third phase began with the adoption of the WRCMSHCP in 2004 and the initiation of land acquisition and long‐term monitoring. Climatically, WRC tended to be in a positive Pacific Decadal Oscillation (PDO) during Time Period 1, leading to greater precipitation in the 1990s and an overall higher precipitation average (Table 2). During the land acquisition (Time Period 2) and monitoring phase (Time Period 3), WRC tended to be negative PDOs with extensive drought (Table 2), particularly during the second period. A latent effect of this split was that we had similar numbers of gnatcatcher observations within each time period, reducing any effect of differing sample sizes.

**Table 2:** (a) Environmental variable means ± standard deviations calculated across the entire WRC (*N* = 74,832 250 m × 250 m cells). See Table 1 for explanation of environmental variables. (b) Environmental variable means ± standard deviations calculated for cells within WRC that were occupied by California gnatcatchers. We present means and ordinary standard deviations based on *n*; however, we use standard errors and degrees of freedom based on *n*_e_. *p <* *.01, 1‐sample *t* test compared to landscape mean (a)

Variable	1980–2012	1980–1997	1998–2003	2004–2012
(a)				
Minimum temperature	—	4.077 ± 2.025	4.068 ± 2.033	3.988 ± 2.13
Maximum temperature	—	25.191 ± 1.953	25.231 ± 1.811	24.974 ± 1.891
Precipitation	—	407.649 ± 104.081	310.061 ± 69.52	359.679 ± 75.023
Elevation	679.811 ± 362.66	—	—	—
Aspect (north facing)	−0.102 ± 0.582	—	—	—
Aspect (east facing)	−0.051 ± 0.598	—	—	—
Slope	8.66 ± 8.101	—	—	—
Coastal sage scrub	—	11.957 ± 0.086	10.102 ± 0.079	9.926 ± 0.078
Chaparral	—	24.538 ± 0.119	23.05 ± 0.117	22.97 ± 0.117
Grassland	—	6.365 ± 0.065	4.887 ± 0.056	4.709 ± 0.055
Agriculture land	—	9.13 ± 0.084	6.251 ± 0.068	5.828 ± 0.066
Developed land	—	2.718 ± 0.033	24.498 ± 0.122	27.529 ± 0.131
High exotic cover	6.63 ± 15.087	—	—	—
Fire history	0.664 ± 1.048	—	—	—
Total nitrogen deposition	9.919 ± 3.013	—	—	—

Variable	1980–1997	1998–2003	2004–2012
(b)			
Minimum temperature	4.674 ± 0.947*	4.376 ± 0.913	4.028 ± 0.75
Maximum temperature	26.401 ± 0.67*	26.599 ± 0.511*	26.352 ± 0.732*
Precipitation	348.292 ± 44.873*	268.872 ± 27.58*	325.789 ± 22.276*
Elevation	451.306 ± 104.34*	467.542 ± 105.11*	519.136 ± 119.52*
Aspect (north facing)	−0.052 ± 0.568	−0.124 ± 0.546	−0.088 ± 0.554
Aspect (east facing)	0.056 ± 0.573	−0.014 ± 0.626	0.008 ± 0.591
Slope	9.739 ± 5.914	9.308 ± 5.258	10.76 ± 6.243
Coastal sage scrub	38.785 ± 32.604*	28.344 ± 29.51*	44.856 ± 28.851*
Chaparral	6.334 ± 17.088*	8.948 ± 19.848*	6.095 ± 15.397*
Grassland	6.055 ± 13.907	3.107 ± 8.295	5.035 ± 10.639
Agriculture land	3.341 ± 12.171*	3.392 ± 10.617	3.141 ± 11.054
Developed land	1.948 ± 6.092	20.474 ± 25.67	9.12 ± 16.066*
High exotic cover	22.417 ± 22.216*	16.854 ± 19.306*	26.906 ± 22.033*
Fire history	0.629 ± 0.891	0.53 ± 0.749	0.935 ± 0.999
Total nitrogen deposition	9.888 ± 1.981	9.21 ± 1.773*	9.196 ± 1.42*
*N* (number of 250 m × 250 m cells)	480	528	554

### 2.1 Partitioned Mahalanobis *D* ^2^ niche modeling

Mahalanobis *D* ^2^ is a niche modeling technique based on the standardized difference between the multivariate mean for environmental variables at locations where a species is detected relative to the values for these same environmental variables at any point in the region being modeled (Clark, Dunn, & Smith, 1993; Rotenberry, Knick, & Dunn, 2002; Rotenberry, Preston, & Knick, 2006). The more similar in environmental conditions a point is to the species’ mean, the smaller the *D* ^2^ and the more “suitable” the habitat at that point is assumed to be. Habitat similarity index (HSI) values are derived from *D* ^2^ values and are generally rescaled to range from 0 to 1 (Clark et al., 1993). An HSI of 1 represents environmental conditions identical to the species’ mean; HSI of 0 represents conditions most dissimilar. *D* ^2^ assumes that at least some environmental variables influencing a species’ distribution have been included in the model, and it performs reasonably well in identifying suitable habitat based on species presence only (Knick and Dyer 1997; Knick & Rotenberry, 1998).

Using eigenvector analysis, Mahalanobis *D* ^2^ can then be partitioned into independent, additive components that represent independent relationships between a species’ distribution and environmental variables (detailed in Rotenberry et al., 2002, 2006). A particular advantage to using partitioned *D* ^2^ is that it focuses on variables that have a relatively low variance across a species’ occurrences; variables maintaining a consistent value where a species occurs (and hence with relatively low variance) are most likely to be associated with factors limiting a species’ distribution, especially compared to those taking on a wider range of values (Dunn & Duncan, 2000; Rotenberry et al., 2002). The number of partitions for a model equals the number of variables included in that model. To choose the appropriate partition that best represented the gnatcatcher's distribution, we calculated the median habitat similarity index for gnatcatcher‐occupied cells for each of the 12 partitions in our model, then chose the partition associated with the largest of those medians as the best partition (Rotenberry et al., 2006). We then used the selected partition to calculate HSI values for every cell in the landscape.

### 2.2 Modeling the California gnatcatcher through time

We selected habitat variables based on previous extensive literature reviews and validation procedures created for WRCMSHCP that are outlined in Preston and Rotenberry (2007) and Preston et al. (2008). There were a total of 12 environmental variables in our models (Table 1). Topographic variables included median values for elevation, aspect, and slope within a 250 m × 250 m grid cell. Topographic variables were calculated from the National Elevation Dataset, 1 arc‐second (30 m) resolution (Gesch, Oimoen, Nelson, Steuck, & Tyler, 2002), and did not change across time periods. Climatic variables included average annual precipitation (mm) and minimum and maximum average temperatures (°C) for the particular time period (PRISM Climate Group 2012). Developed land, defined as urban landscapes in the National Landcover Database (NLD; USGS 2015), was calculated as a percentage of developed land within each 250 m × 250 m grid cell. Percentages were obtained for the years 1992 (used for Time Period 1), 2002 (used for Time Period 2), and 2012 (used for Time Period 3). Vegetation variables (percent CSS, Chaparral, and Grassland habitats) and percent agricultural lands were calculated by taking 2004 aerial measurements from vegetation maps of WRC at a 6‐m resolution resampled to 30 m (Klein & Evens, 2005). Vegetation percent covers were then proportionally adjusted for each time period for each cell based on the percentage changes of land development from the NLD within each cell by taking the amount of developed land calculated during the 2004 aerial measurements and determining the difference for the 1992, 2002, and 2012 years from the NLD. We then proportionally adjusted percent vegetation covers based on the change in land development. For example, if the percent cover of vegetation was 75%, 25%, 0% for CSS, Chaparral, and Grassland habitats, respectively, in the 2004 aerial assessments and we observed a change in developed land from the 2004 aerial survey of +20%, we subtracted 15% coverage of CSS habitats and 5% coverage of Chaparral habitat. If a proportional change decreased a habitat below zero, we assumed a zero percent cover of that habitat; if a proportional change increased a habitat over 100, we assumed a 100% cover of that habitat. We note that this calculation assumes that changes in development affect each vegetation type to the same degree. We then calculated the WRC‐wide mean and standard deviation of all 12 environmental variables for each time period (Table 2).

We modeled gnatcatcher habitat within each time period using the partitioned Mahalanobis *D* ^2^ applied to the 12 environmental variables for each period. We modeled each time period separately to account for any potential changes in the variables that might limit gnatcatcher distribution. Calculations were carried out in SAS (SAS Institute 2001) using SAS code from Rotenberry et al. (2006). We then validated the models quantitatively using the *evaluate()* command in the {dismo} package in R (Core Team, 2012). This analyses cross‐validates models with presence only or presence/absence data. Given a vector of presences and a vector of absences (or in our case pseudoabsences generated by *evaluate()*), and a vector of HSI values, confusion matrices are computed (for varying thresholds), and model evaluation statistics (AUC values) are computed for each confusion matrix/threshold. Models are considered robust if AUC is ≥0.70 (Fielding & Bell, 1997).

Once we validated models, within each time period, we calculated HSIs for every cell in the landscape. For visualization, using ArcGIS (ESRI 2012) we mapped each cell as either highly suitable (HSI ≥ 0.66), moderately suitable (0.33 < HSI < 0.66), or minimally suitable (HSI ≤ 0.33), following categories defined by Preston and Rotenberry (2007) and Preston et al. (2008). We then simplified our HSI categories into habitat that was either suitable (HSI ≥ 0.66) or unsuitable (HSI < 0.66) and created suitability change maps representing each cell's suitability transition from the first to second time period and the second to third time period. Thus, we had four transition categories: (1) cells that maintained high habitat suitability (2) cells that remained unsuitable, (3) cells that lost suitability, and (4) cells that gained suitability. Finally, we calculated the number of cells that were highly suitable or unsuitable in each time period in order to better understand the amount of area that gained or lost suitability.

### 2.3 Relating environmental variables to suitability changes within grid cells

For environmental analyses, we included three additional environmental variables that are known stressors within WRC: exotic plant cover, nitrogen deposition, and fire frequency (Table 1). Nitrogen deposition values were calculated on a 4‐km grid using the Community Multiscale Air Quality model (Tonnesen, Wang, Omary, & Chien, 2007). Values represent total annual N deposition (NO_3_^−^ and NH_4_^+^) from 2002. Exotic plant cover was the amount of CSS habitat with >25% exotic cover within a 250‐m × 250‐m grid cell that was measured during aerial surveys in 2004 (Klein & Evens, 2005). Fire history was the number of fires that occurred over a 60‐year time period (1943–2003; data sourced from USGS 2008 and California Department of Forestry 2008). We did not include these in our model as we only had one time period measurement for each grid cell, but these variables are not static.

To aid in our assessment of how the environmental variables collectively related to suitability, we calculated means for the 15 environmental variables for suitable grid cells (HSI ≥ 0.66) and for unsuitable grid cells (HSI < 0.66) for each time period. We analyzed differences in average value of each environmental variable using Student *t* tests comparing the two classes. However, the presence of positive spatial autocorrelation apparent in our landscape (i.e., points physically closer together are more likely to share similar values for a variable than points further apart) creates a lack of statistical independence among cells and thus invalidates most normal tests of statistical significance (e.g., Dale & Fortin, 2002). Intuitively, because some sample points are not fully independent of one another, our “effective” sample size is less than the nominal sample size (*n*), increasing the chance of Type I error. To compensate, we calculated an effective sample size (*n*_e_) following Griffith (2005; eqn. 3); for each test, we performed assuming a very high level of spatial dependency (e.g., ρ = 0.8, for a simple autoregressive model of *y* = ρ**C**y + ε, where y is a variable of interest, **C** is a matrix of spatial weights [e.g., inverse distances], and ε represents error). Although this will lead us to underestimate statistical significance for variables with low spatial autocorrelation, this approach will provide some assurance that any apparently statistically significant results we observe are indeed significant. Sample means and standard deviations are calculated in the usual way, then *n*_e_ is used instead of *n* in calculating standard errors and, hence, *t*‐statistics (Dale & Fortin, 2002). We also compared environmental variable means using the method described above between habitat that gained or lost suitability through time based on protection level.

### 2.4 Assessing the effectiveness of prioritized conservation lands

Conservation lands included properties within WRC owned, managed, or maintained by public agencies for conservation purposes along with established habitat reserves for the protection of species covered by the MSHCP (Figure 2b). To assess the effectiveness of conservation lands in protecting gnatcatcher habitat, we calculated the number of cells that gained or lost suitable habitat or remained suitable between each time period based on whether the grid cell was considered protected or not. We then calculated percent change in gained and lost habitat through time based on protection status.

## 3 RESULTS

### 3.1 Modeling the California gnatcatcher through time

For the WRC study area as a whole, temperatures across time periods only varied about 0.2°C for maxima and less than 0.1°C for minima (Table 2a). On the other hand, precipitation varied substantially, declining nearly 100 mm between the first and second periods, then increasing by nearly 50 mm between the second and third. All of the land cover classes changed substantially through time: coastal sage scrub declined 17% between the first and third periods, chaparral −6%, grassland −26%, and agriculture −36%. The most dramatic change was in developed land, which increased over 900%, most of which occurred between the first and second periods (Tables 2a,b; Fig. S1).

There were 480, 528, and 554 unique grid cells where gnatcatchers were observed for the first, second, and third time periods, respectively. Cells occupied by gnatcatchers differed significantly from the landscape as a whole with respect to a number of environmental variables (Table 2b). Occupied cells were consistently warmer and drier and at lower elevations. They also contained more coastal sage scrub but less chaparral, with higher exotic cover and generally higher total nitrogen deposition. They had less than average agriculture during the first period (when agriculture was highest) and less than average development during the third period (when development was highest). Characteristics of occupied cells were also largely consistent in composition from one year to the next, differing significantly among periods only in precipitation and development, the latter only between the first and second periods (Table 2b).

The niche models were considered robust with AUC values of 0.84, 0.83, and 0.86 for the first, second, and third time periods, respectively, and we selected partition 1, 3, and 3 for the three time periods, respectively, as these yielded the highest average HSI values for gnatcatcher‐occupied cells. Highly suitable habitat (HSI ≥ 0.66) was consistently warmer and drier and occurred at a lower elevation than less suitable habitat (Table 3); however, during each time period, more suitable habitat became cooler and higher in elevation, particularly comparing the third period to the first. Suitable maximum temperature varied little among time periods, and the relationship between mean highly suitable HSI and precipitation appeared to fluctuate with precipitation's natural variation. In one period, highly suitable habitat was significantly more south‐facing, and in two periods, significantly more west‐facing (Table 3). Highly suitable habitat consistently had substantially more coastal sage scrub, much less chaparral, less grassland and agriculture, and higher exotic cover (Table 3). In one period, it was associated with more historical fires and less deposited nitrogen, but not in the other two (Table 3). Although high HSI cells were associated with relatively more development than low HSI in the second time period, they had a large negative association in the third period (Table 3). Overall, results from comparing high‐ versus low‐suitability cells largely paralleled those comparing occupied to unoccupied cells.

**Table 3:** Environmental means ± standard deviation for highly suitable (HSI ≥ 0.66) and less suitable (HSI < 0.66) grid cells for three time periods. Raw (*N*) and effective (*N*_e_) sample sizes in parentheses. We present means and ordinary standard deviations based on *n*; however, we use standard errors and degrees of freedom based on *n*_e_. *p <* *.01, 1‐sample *t* test compared to unsuitable mean

Environmental variable	1980–1997		1998–2003		2004–2012	
	Suitable *(N = 9,258, N*_e_ * = 788)*	Unsuitable *(N = 65,574)*	Suitable *(N = 8,177, N*_e_ * = 697)*	Unsuitable *(N = 66,655)*	Suitable *(N = 4,860, N*_e_ * = 415)*	Unsuitable *(N = 66,655)*
Minimum temperature	4.264 ± 0.866*	4.051 ± 2.139	4.239 ± 0.841*	4.047 ± 2.133	3.766 ± 0.696*	4.004 ± 2.194
Maximum temperature	26.548 ± 0.481*	24.999 ± 2.006	26.595 ± 0.543*	25.064 ± 1.841	26.144 ± 0.882*	24.893 ± 1.915
Precipitation	340.35 ± 30.69*	417.15 ± 107.24	271.97 ± 25.88*	314.73 ± 71.72	329.30 ± 24.01*	361.79 ± 76.88
Elevation	465.73 ± 79.57*	710.03 ± 376.58	473.92 ± 116.00*	705.07 ± 374.39	573.22 ± 153.83*	687.22 ± 371.72
Aspect (north facing)	−0.062 ± 0.577	−0.050 ± 0.605	−0.165 ± 0.537*	−0.037 ± 0.604	−0.091 ± 0.533	−0.048 ± 0.602
Aspect (east facing)	−0.088 ± 0.481	−0.104 ± 0.591	−0.164 ± 0.488*	−0.095 ± 0.592	−0.189 ± 0.521*	−0.096 ± 0.586
Slope	7.491 ± 5.677*	8.826 ± 8.377	6.447 ± 5.274*	8.932 ± 8.342	9.158 ± 6.051	8.626 ± 8.223
Coastal sage scrub	30.368 ± 8.660*	9.357 ± 21.367	16.406 ± 23.357*	9.329 ± 21.244	33.168 ± 29.295*	8.311 ± 19.787
Chaparral	3.397 ± 0.866*	27.522 ± 33.701	7.457 ± 15.975*	24.963 ± 32.871	10.145 ± 17.303*	23.861 ± 32.512
Grassland	3.510 ± 7.120*	6.768 ± 18.856	1.189 ± 3.233*	5.340 ± 16.272	3.029 ± 5.958*	4.826 ± 15.574
Agriculture land	1.547 ± 4.811*	10.200 ± 24.252	1.860 ± 5.184*	6.789 ± 19.608	3.480 ± 8.094*	5.991 ± 18.527
Developed land	1.267 ± 2.790*	2.923 ± 9.635	31.144 ± 27.533*	23.683 ± 33.961	11.159 ± 13.567*	28.666 ± 36.742
High exotic cover	20.074 ± 21.745*	4.731 ± 12.803	14.330 ± 19.963*	5.685 ± 14.089	23.906 ± 23.645*	5.430 ± 13.507
Fire history	0.700 ± 1.155	0.658 ± 1.039	0.589 ± 0.941	0.673 ± 1.060	0.954 ± 1.297*	0.643 ± 1.026
Total nitrogen deposition	9.863 ± 2.021	9.926 ± 3.131	9.929 ± 3.089	9.917 ± 3.004	9.026 ± 1.826*	9.981 ± 3.069

### 3.2 Changing habitat suitability through time

**Figure 3:**
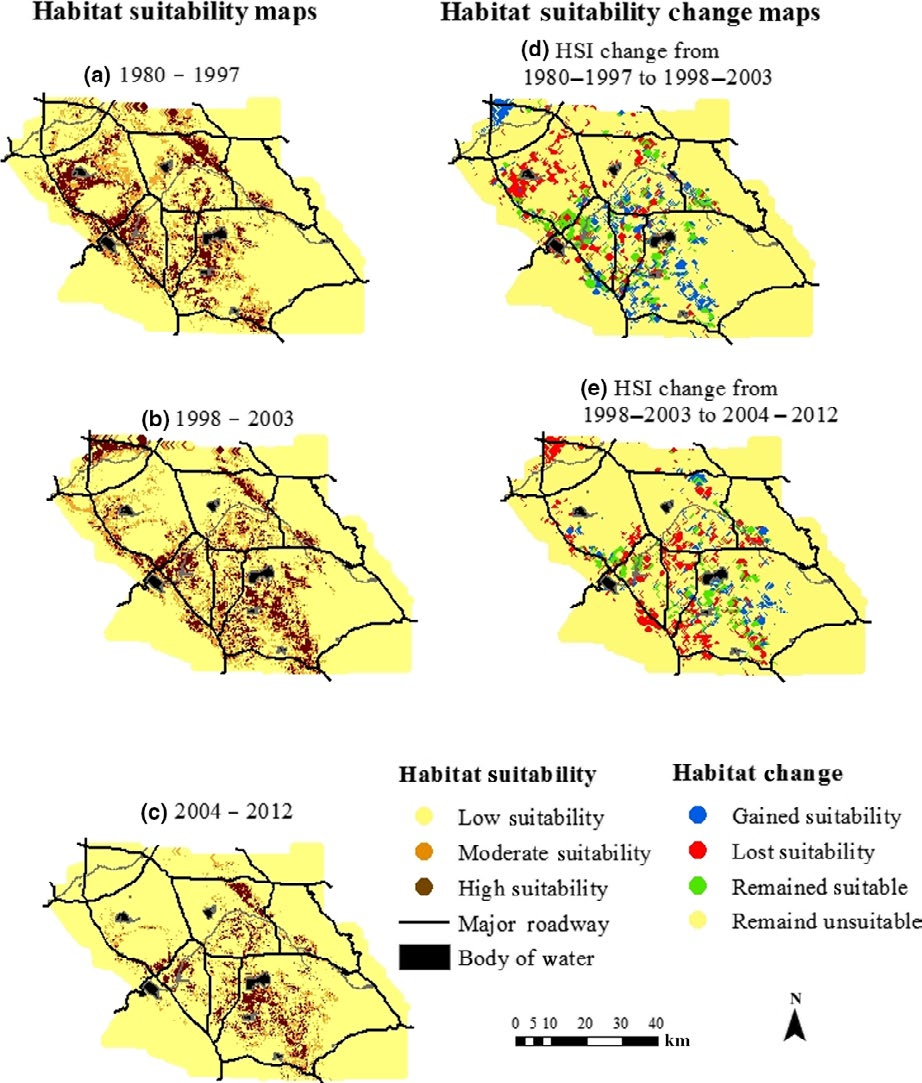
(a–c) Habitat suitability maps of the California gnatcatcher for the following time period: (a) 1980–1997, (b) 1998–2003, and (c) 2004–2012, and habitat suitability change maps of the California gnatcatcher between the following time period: (d) 1980–1997 and 1998–2003 and (e) 1998–2003 and 2004–2012

The gnatcatcher lost 11.7% and 40.6% of cells with highly suitable habitat within WRC between time period transitions (Table 4a,b). Visually, we can see the reduction in highly suitable habitat through time (Figure 3a–c), and its shift toward the southeast section of the study area (Figure 3d,e). The largest numbers of grid cells regardless of their starting and middle suitability transition states were those considered unsuitable in the third time period (Fig. S2). Of the cells that were unsuitable during 1980–1997 and became suitable during 1998–2003, 76% lost their suitability during 2004–2012 (Fig. S2). Only 1,918 grid cells (20.7%) maintained high suitability through all time periods out of the initial 9,258 grid cells that were suitable in the first time period (Fig. S2).

**Table 4:** (a) Number of grid cells that were highly suitable based on whether cell was within protected conservation area and (b) number of grid cells that gained or lost high suitability based on whether cell was within protected conservation area

Period	Protected	Unprotected	Total
(a)			
1980–1997	4,610	4,648	9,258
1998–2003	3,802	4,375	8,177
2004–2012	3,202	1,658	4,860

Transition period	Protected			Unprotected			Total		
	Gain	Loss	Net change	Gain	Loss	Net change	Gain	Loss	Net change
(b)									
1980–1997 to 1998–2003	1,635	2,443	−17.5%	2,311	2,584	−15.8%	3,946	5,027	11.7%
1998–2003 to 2004–2012	1,318	1,918	−6.2%	693	3,410	−62.1%	2,011	5,328	40.6%

### 3.3 Assessing the effectiveness of conservation lands

During the first transition period, the number of highly suitable cells declined 18% within protected areas, but only 6% in unprotected ones (Table 4). However, during the second transition period, the number of highly suitable cells declined 16% within protected areas, but 62% outside protection. Overall, there was a 31% decline in protected highly suitable habitat cells, and 64% decline outside protection.

Several environmental variables differed significantly among cells that changed in suitability within protected areas from Time Period 1 to Time Period 2. Compared to losses, gains in suitability within protected areas were characterized by lower minimum temperatures, less precipitation, less agriculture, and less chaparral (Table 5). Unprotected cells that gained compared to losing suitability were also dryer and had less agriculture, but had more coastal sage scrub. During the second transition period, changes in suitability both inside and outside protected areas were similarly driven by climate; compared to losses, gains were associated with warmer minima, cooler maxima, and less precipitation, and gains were associated with less development and more coastal sage scrub inside and out (Table 5). Cells that gained suitability in protected areas had more chaparral, whereas those outside protection had less agriculture.

**Table 5:** Mean difference (±standard deviation) of grid cells that gained or lost high suitability based on whether cell was within protected conservation area for the transitions between 1980–1997 to 1998–2003, and 1998–2003 to 2004–2012. We present means and ordinary standard deviations based on *n*; however, we use standard errors and degrees of freedom based on *n*_e_. *p <* *.01, 2‐sample *t* test comparing mean gain to mean loss

	Protected			Unprotected		
	Mean change (±*SD*)		Gain compared to loss	Mean change (±*SD*)		Gain compared to loss
	Gained	Lost		Gained	Lost	
1980–1997 to 1998–2003						
Minimum temperature	−0.192 ± 0.511	0.175 ± 0.248	−0.368*	0.289 ± 0.358	0.286 ± 0.263	0.003
Maximum temperature	−0.018 ± 0.114	−0.030 ± 0.113	0.012	−0.046 ± 0.240	−0.009 ± 0.149	0.037
Precipitation	−90.887 ± 18.025	−74.133 ± 14.459	−16.753*	−82.785 ± 20.149	−72.948 ± 15.599	−9.837*
Development	21.243 ± 23.615	12.418 ± 23.371	8.825	44.205 ± 24.059	46.817 ± 36.987	2.612
Grassland	−1.607 ± 6.996	−0.834 ± 3.020	−0.773	−2.866 ± 8.945	−1.138 ± 3.753	−1.727
Agriculture	−6.923 ± 15.705	−0.253 ± 1.624	−6.671*	−13.620 ± 18.688	−0.705 ± 2.805	−12.915*
Coastal sage scrub	−1.677 ± 5.421	−4.392 ± 9.790	2.714	−1.998 ± 6.498	−8.423 ± 15.618	6.425*
Chaparral	−2.884 ± 6.060	−0.597 ± 2.874	−2.287*	−1.012 ± 5.150	−0.795 ± 3.730	−0.217
*n*_e_	140	209		198	221	
*n*	1,635	2,443		2,311	2,584	
1998–2003 to 2004–2012						
Minimum temperature	0.060 ± 0.241	−0.051 ± 0.220	0.111*	−0.021 ± 0.207	−0.086 ± 0.154	0.065*
Maximum temperature	−0.279 ± 0.107	−0.188 ± 0.110	−0.091*	−0.276 ± 0.110	−0.162 ± 0.130	−0.115*
Precipitation	51.293 ± 9.354	54.675 ± 13.070	−3.382*	50.619 ± 9.003	54.190 ± 14.129	−3.571*
Development	0.397 ± 3.795	4.105 ± 13.540	−3.709*	1.841 ± 6.633	10.488 ± 19.319	−8.647*
Grassland	−0.183 ± 2.511	−0.062 ± 0.749	−0.122	−0.261 ± 2.437	−0.116 ± 1.016	−0.145
Agriculture	−0.166 ± 2.405	−0.172 ± 1.558	0.006	−1.387 ± 5.750	−0.513 ± 2.764	−0.874*
Coastal sage scrub	−0.034 ± 0.681	−0.376 ± 4.031	0.342*	−0.119 ± 1.208	−0.698 ± 4.594	0.579*
Chaparral	−0.003 ± 0.107	−0.133 ± 1.851	0.130*	−0.026 ± 0.490	−0.058 ± 0.824	0.032
*n*_e_	113	164		60	291	
*n*	1,318	1,918		693	3,410	

## 4 DISCUSSION

Our study shows the utility of using ecological niche models for understanding how environmental variables are related to changes in habitat suitability through time. Further, we provide evidence for the importance of creating habitat plans that maintain adequate protected lands. Our data described a species’ changing distribution in response to environmental change and provide a background for creating and adapting conservation plans to begin to accommodate future responses to environmental change. Over the past four decades, the gnatcatcher has lost large amounts of suitable habitat within WRC especially between the second and third time periods. The change in suitable habitat was driven mainly by landscape development across CSS habitat, but also yielded some changes in climate‐related associations.

Gnatcatchers occupied cells that varied less (both through time and within occupied cells) for maximum temperature than minimum temperature, and we did not find a consistent temporal trend or significant difference in maximum temperature between cells that gained or lost suitability. However, we found significant differences between mean minimum temperatures among cells that gained or lost suitability. Thus, when predicting future geographic responses to temperature changes for the gnatcatcher, it is crucial to allow flexibility (plasticity) with minimum temperature, but restrict maximum temperature for future scenarios. We note that based on the changing response curve to the relationship between habitat suitability changes and the environmental variables through time, the gnatcatcher may also be adapting to environmental change or have a high plasticity for those variables. Additional studies are needed to disentangle adaptation from plasticity in the gnatcatcher's response to environmental change.

Although the loss of suitable cells within protected areas outpaced gains, the rate of loss was manifestly less than that outside. Thus, it is clear that gnatcatcher habitat is better maintained within protected areas than outside, but only insofar as it has experienced a less rapid decline inside than out. Changes in both climate and land use/land cover were associated with changes in habitat suitability inside and outside protected areas, but no easily interpretable differences between the two were apparent. It seems therefore that processes that modify suitability are generally the same outside versus inside, but are more extensive outside protected areas.

The mean minimum temperature of suitable habitat declined slightly through time; the lower minimum temperature is likely to be a latent effect of the movement toward higher elevation. Although this is consistent with literature showing that as temperatures rise due to climate change, bird species are known move up in elevation to stay within their physiological tolerances (Tingley, Monahan, Beissinger, & Moritz, 2009), it seems more likely that movement toward higher elevation may be a by‐product of displacement as lower elevational landscapes are developed with concomitant loss of CSS. Both maxima and minima temperatures remained within the known temperature constraints of the gnatcatcher (Mock 1998). Interestingly, lower temperatures at higher elevations are likely to pose a stronger distributional limit to gnatcatchers than higher temperatures at lower elevations. Range limits in otherwise suitable vegetation types in southern California appear to be associated with average January minima of about 2.5°C (Mock 1998), only about 2°C below what we observed. On the other hand, summer maxima in gnatcatcher‐occupied areas in Baja California Sur routinely exceed 35°C (Hijmans, Cameron, Parra, Jones, & Jarvis, 2005), well above those observed in our study area.

The relationships among the variables we considered in our analyses are complex. One important feature is that things such as topography, nitrogen deposition, fire history, and agriculture/development exert their effects on gnatcatchers only *indirectly*, via their direct effects on vegetation type. Gnatcatchers do not seem to be edge‐sensitive (Bolger, Scott, & Rotenberry, 1997), and hence, effects of agriculture and development are mainly manifest through habitat loss. Nevertheless, even indirect effects can be rapid and profound, mediated through sudden impacts of land‐use change and/or fire on vegetation type, including complete replacement of one land cover type by another. A further complication from a modeling and change‐detection perspective is that topography is a fixed effect (no temporal variance, at least over relevant time scales), yet is a strong driver of local and regional patterns of climate and land use. Indeed, in our analysis, increasing elevation appears to be associated with changes in habitat suitability, but this is most like an expression of the fact that most development and agriculture have occurred below 600 m, where most CSS historically occurred as well. And although patterns of precipitation and temperature may vary annually, absent persistent trends vegetation types are likely to change slowly if at all. Should trends persist, however, climate change's direct and indirect impacts on gnatcatchers may both be profound (Preston et al., 2008).

Two things are shifting through time: the distribution of values of environmental variables (e.g., precipitation, temperature, CSS, development) and the distribution of California gnatcatchers in response to some of those variables. Although the three partitioned Mahalanobis *D* ^2^ niche models that capture bird–environment relationships were fundamentally similar, they differed in some attributes between periods. We do not believe that these represent “niche shifts” or local adaptive changes in gnatcatcher habitat relationships, but rather suggest that the nature of the limits to the distribution of the species in this region may shift between periods, compounded by lags in the response of gnatcatchers to environmental change (i.e., “ghosts of habitats past”; Knick & Rotenberry, 2000). Such “ghosts” are most likely to occur when largely sedentary, permanent resident species such as the California gnatcatcher (Atwood & Bontrager, 2001) are confronted with rapid (but nonlethal) habitat changes. This is best indicated by the relationship between gnatcatchers, CSS, and development. Development burgeoned during the middle time period, most often at the expense of CSS. In the first period, both occupied cells and the niche model showed a strong positive association of birds with CSS but little association with development, which was comparatively sparse throughout the study area. In the second period, the association with CSS appeared weaker whereas development was now abundant in occupied cells and indeed higher in cells deemed suitable than in unsuitable ones. In the third period, development had increased comparatively less, and the positive association of gnatcatchers with CSS and negative one with development was again manifest. During periods of high development, the complexity of environmental factors became evident. Vegetation types, precipitation, and exotic species coverage all had varying positive or negative associations with gained and lost habitat depending on the time period (the first and third time periods tended to have one relationship with grid cells that gained and lost suitability while the second time period that was undergoing high development had the opposite relationship). Together, these suggest that the interacting effects of some environmental variables may alter the influence of other environmental variables depending on the time period and its current stressors. Multiple environmental stressors operating at different scales influence population dynamics and may change a species’ distribution (Preston et al., 2012). Recent studies by Swab et al. (2012) and Preston et al. (2012) found that predicted range shifts induced by climate change were only part of the threat for the future outcome of two plant species, and other environmental stressors may play an equal or larger or interactive role in the survival of a species.

Based on bioclimate modeling of species’ suitable climate space (based on mean temperatures and precipitation, particularly those variables serving as proxies for extreme conditions), Bateman et al. (2015) demonstrated significant geographic shifts in potential breeding distribution of land birds, with an average velocity of 1.27 km/yr. However, they also implicated human‐imposed land‐use changes in influencing potential species richness in a region, noting that areas that had become increasingly suitable for birds due to changing climate were often those attractive to humans for agriculture and development. They suggest that many areas might have supported more breeding bird species had the landscape not been altered. In our study, we detected a relatively faint signal of changing climate, but a strong one of development and other changes in land cover types, with follow‐on effects on the amount and distribution of highly suitable habitat for gnatcatchers. Thus, although direct and indirect effects of climate change may be long‐term drivers of changing habitat suitability for gnatcatchers in the WRC MSHCP planning area (e.g., Preston et al., 2008), in the near term, regional land‐use practices may have a greater effect. Projections of future climate appropriately down‐scaled to the regional planning area may permit more detailed modeling of future higher (and lower) quality habitat, perhaps providing a template for directing development to areas where it would not preclude protection of suitable habitat yet to come.

Habitat loss has been the greatest threat to biodiversity, at least in the near term (Brooks et al., 2002; Groom & Grubb, 2006; Hanski, 2005). This factor was evident in the impact of expanding development and conversion of CSS within southern California on the California gnatcatcher. While the gnatcatcher lost substantial highly suitable habitat throughout the region, our study found that land outside of conservation protection by the WRCMSHCP lost more suitable habitat than habitat within conservation lands over the course of our study. These findings emphasize the importance of providing protected areas beyond the present distributions, as these may mitigate at least some of the negative effects of environmental changes that do not respect political boundaries. Changing climate and the development of land due to lack of protection outside the initial critical habitat may be crucial to the long‐term protection of the gnatcatcher and other species.

While southern California is considered a biodiversity hotspot, the remaining CSS especially is also experiencing a multitude of anthropogenic stressors that differentially affect an already patchy habitat type. Because of this, prioritizing future conservation lands is critical to successfully manage habitat for plant and animal populations. During our study period, WRC adopted a multiple habitat and species conservation plan in 2004 with a goal of conserving 146 sensitive plant and animal species and their habitats (Western Riverside County Regional Conservation Authority 2017). Based on the trends of the nonprotected lands losing suitability in the most recent period, our data suggest that the gnatcatcher would have lost even larger amounts of suitable habitat without the prioritization of conservation lands. Our study emphasizes the importance of creating habitat conservation plans and prioritizing conservation lands as well as protecting project future habitats are crucial maintain populations during periods of high environmental stress.

## CONFLICT OF INTEREST

None declared.

## AUTHORS CONTRIBUTION

Dr. Heather Hulton VanTassel was the primary researcher of this study and writer of the manuscript. Dr. Michael D. Bell assisted with analyses, provided invaluable feedback, and edited manuscript. Dr. John Rotenberry provided statistical expertise, SAS code for model runs, and invaluable insight into the research while largely contributing to the written manuscript edits. Robert Johnson compiled all data sets required for this research while providing edits of the manuscript. Dr. Michael F. Allen advised Dr. Hulton VanTassel throughout the research study while providing invaluable insights into the analyses and manuscript edits.
