# Supplementary figures and images for: Environmental change, shifting distributions, and habitat conservation plans: A case study of the California gnatcatcher
Source: Ecol Evol. 2017 Oct 28;7(23):10326–38. doi: 10.1002/ece3.3482 (PMC5723624; doi:10.1002/ece3.3482)

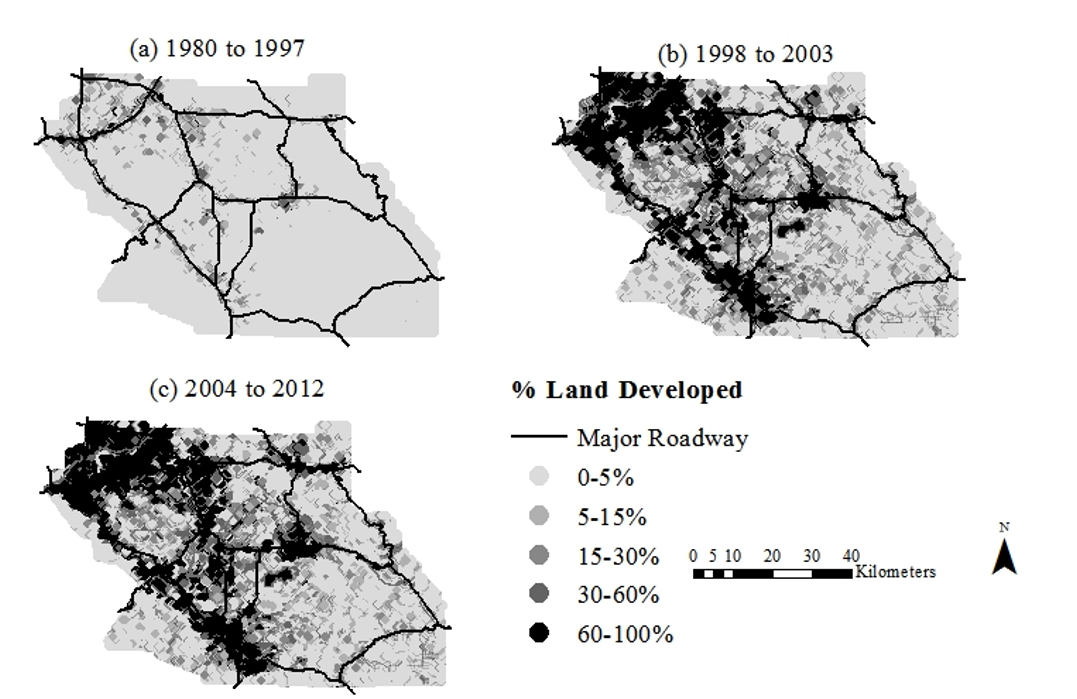

Supplement: Supplementary file 1 [file ECE3-7-10326-s001.tif]

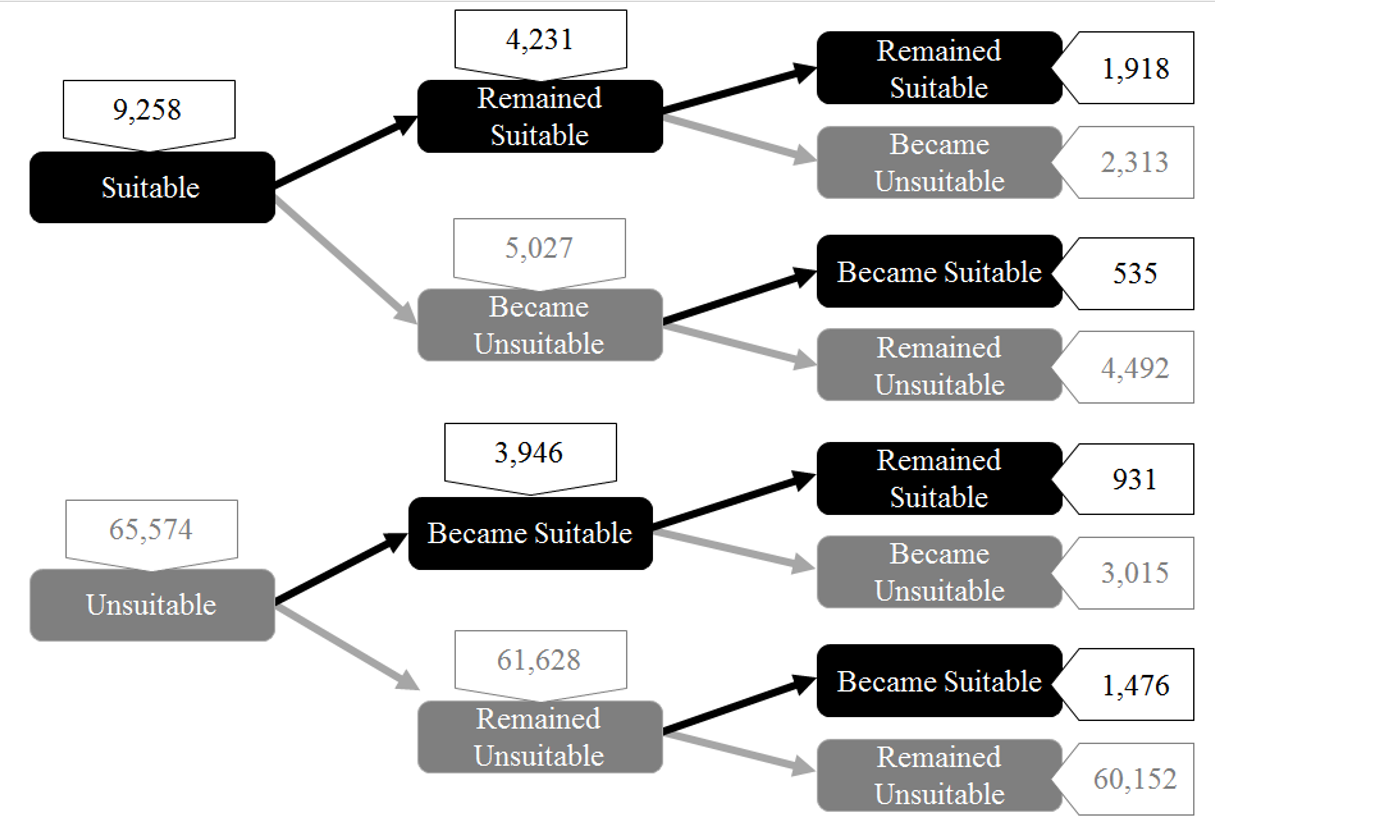

Supplement: Supplementary file 2 [file ECE3-7-10326-s002.tif]
