# Supplementary material for: Environmental change, shifting distributions, and habitat conservation plans: A case study of the California gnatcatcher
Source: Ecol Evol. 2017 Oct 28;7(23):10326–38. doi: 10.1002/ece3.3482 (PMC5723624; doi:10.1002/ece3.3482)
Supplement: Supplementary file 3 [file ECE3-7-10326-s003.docx]

**Supporting Information:**

**Figure S1:**  Percent developed land within WRC in 1992, 2002, 2012 assigned to the time periods (a) 1980-1997, (b) 1998-2003, (c) 2004-2012.

**Figure S2**: Number of grid cells based on state of suitability through time.
